# GWAS and co-expression network combination uncovers multigenes with close linkage effects on the oleic acid content accumulation in *Brassica napus*

**DOI:** 10.1186/s12864-020-6711-0

**Published:** 2020-04-23

**Authors:** Min Yao, Mei Guan, Zhenqian Zhang, Qiuping Zhang, Yixin Cui, Hao Chen, Wei Liu, Habib U. Jan, Kai P. Voss-Fels, Christian R. Werner, Xin He, Zhongsong Liu, Chunyun Guan, Rod J. Snowdon, Wei Hua, Lunwen Qian

**Affiliations:** 1 Collaborative Innovation Center of Grain and Oil Crops in South China, Hunan Agricultural University, Changsha, 410128 China; 2 College of Horticulture and Landscape Architecture, Southwest University, Chongqing, 400715 China; 3 Precision Medicine Lab, Rehman Medical Institute (RMI), Phase 5, Hayatabad, Peshawar, 25000 Pakistan; 4 Queensland Alliance for Agriculture and Food Innovation, The University of Queensland, St Lucia, QLD Australia; 5 The Roslin Institute University of Edinburgh Easter Bush Research Centre Midlothian, Edinburgh, EH25 9RG UK; 6 Department of Plant Breeding, IFZ Research Centre for Biosystems, Land Use and Nutrition, Justus Liebig University, Heinrich-Buff-Ring 26-32, 35392 Giessen, Germany; 7 Oil Crops Research Institute of the Chinese Academy of Agricultural Sciences, Key Laboratory of Biology and Genetic Improvement of Oil Crops, Ministry of Agriculture, Wuhan, 430062 China

**Keywords:** Oleic acid, GWAS, Haplotype, Co-expression network, *Brassica napus*

## Abstract

**Background:**

Strong artificial and natural selection causes the formation of highly conserved haplotypes that harbor agronomically important genes. GWAS combination with haplotype analysis has evolved as an effective method to dissect the genetic architecture of complex traits in crop species.

**Results:**

We used the 60 K *Brassica* Infinium SNP array to perform a genome-wide analysis of haplotype blocks associated with oleic acid (C18:1) in rapeseed. Six haplotype regions were identified as significantly associated with oleic acid (C18:1) that mapped to chromosomes A02, A07, A08, C01, C02, and C03. Additionally, whole-genome sequencing of 50 rapeseed accessions revealed t*hree genes (BnmtACP2-A02, BnABCI13-A02 and BnECI1-A02) in the* A02 chromosome haplotype region and two *genes* (*BnFAD8-C02 and BnSDP1-C02) in the* C02 chromosome haplotype region that were closely linked to oleic acid content phenotypic variation. Moreover, the co-expression network analysis uncovered candidate genes from these two different haplotype regions with potential regulatory interrelationships with oleic acid content accumulation.

**Conclusions:**

Our results suggest that several candidate genes are closely linked, which provides us with an opportunity to develop functional haplotype markers for the improvement of the oleic acid content in rapeseed.

## Background

Oilseed rape (*Brassica napus L.*) is an allotetraploid species with 2*n* = 38 chromosomes and two genomes (AA derived from *B. rapa* and CC from *B. oleracea*). It is the most important source of edible vegetable oil and protein-rich meal in China. Rapeseed oil is similar to other vegetable oils. Its fatty acid composition is the key trait involved in its utilization mode and range [1]. The proportions of the three major unsaturated fatty acids in rapeseed oil are 60% oleic acid [C18:1], 4% palmitic acid [C16:0] and 2% stearic acid [C18:0]. Furthermore, oleic acid has been recognized as having health benefits, including effectiveness in reducing overall cholesterol levels, as well as anti-arteriosclerosis and cardiovascular protective effects; therefore, a higher oleic acid content level in seed oil is a desirable trait. Menendez et al. [2] suggested that the anti-cancerous and heart-protective properties of the Mediterranean diet could also be attributed to 18:1. Pinzi et al. [3] suggested that high oleic acid oil was an ideal raw material for biodiesel production. High oleic acid oil can be heated to a higher temperature without smoking, which makes it more suitable as a cooking oil. Therefore, further improvement of the oleic acid oil content has become a primary goal of rapeseed breeding.

In plants, fatty acid biosynthesis is a very complicated process. Li-Beisson et al. [4] reported more than 120 enzymatic reactions and at least 600 genes were involved in acyl-lipid metabolism process in *Arabidopsis*. Fatty acid biosynthetic pathways in rapeseed are considered quantitative traits regulated by QTLs. Wang et al. [5] detected 72 individual QTLs and a large number of pairs of epistatic interactions associated with the content of 10 different fatty acids. For the oleic acid (C18:1) content, the major QTLs are mainly distributed across A03, A05, A08, C03 and C08 [6–11]. Since the advent of highly efficient genotyping technologies, the genome-wide association study (GWAS) has become the method of choice for dissection of complex plant traits. For example, a GWAS detected more than 100 single nucleotide polymorphisms (SNPs) in significant associations with the oleic acid content on chromosomes A06, A08, A09, C01, C3, C04, C08 and C09 [12]. Guan et al. [13] identified 95 candidate genes involved in fatty acid biosynthesis in the whole genome using a GWAS in combination with RT-qPCR analysis.

Strong selection can cause multigene close linkages in genome regions related to target trait phenotypic variation. Recently, some haplotype-based GWAS detected haplotype regions containing several causal genes related to phenotypic variation in crops. For example, a GWAS performed with the Illumina 90 k SNP Infinium array to check a haplotype region (at 137.1 and 143.5 cM) that contained candidate genes related to root growth on chromosome 5B in wheat [14]. Qian et al. [15] identified a haplotype carrying candidate genes *BnTOC159* and BnaA02g20650D, which were significantly associated with the leaf chlorophyll content on chromosome A02 in rapeseed. A genome-wide haplotype association analysis detected haplotypes containing two candidate genes (glyma12g075700 and glyma12g075600) that showed significant associations with the seed weight and seed yield in soybean [16]. Although the haplotype regions containing several causal genes that are significantly associated with the target trait can be detected, however, little is known if these putative genes have a potential relationship with the haplotype regions.

Gene function can be established through reverse genetic approaches in many crops, and co-expression networks have been shown to be a powerful tool for the rapid prediction of potential functional links between genes. Recent studies have suggested that a GWAS in combination with a co-expression network is a powerful tool to identify genes related to phenotypic variation in maize and *Populus* [17, 18]. This method will also permit analysis of relationships among genes with significant associations in a haplotype region.

In our study, we perform a *GWAS of haplotype blocks* and identify six haplotype blocks carrying causal genes associated with the seed oleic acid content in a diverse *B. napus* population*.* In particular, our objectives were to uncover several candidate genes in close linkage due to strong selection and to identify a potential molecular network regulated in the accumulation of oleic acid content by whole-genome sequencing of 50 accessions and gene co-expression. Our results will help in developing functional haplotype makers to further improve oleic acid content in *B. napus.*

## Results

### Oleic acid content variation and correlations

**Fig. 1:**
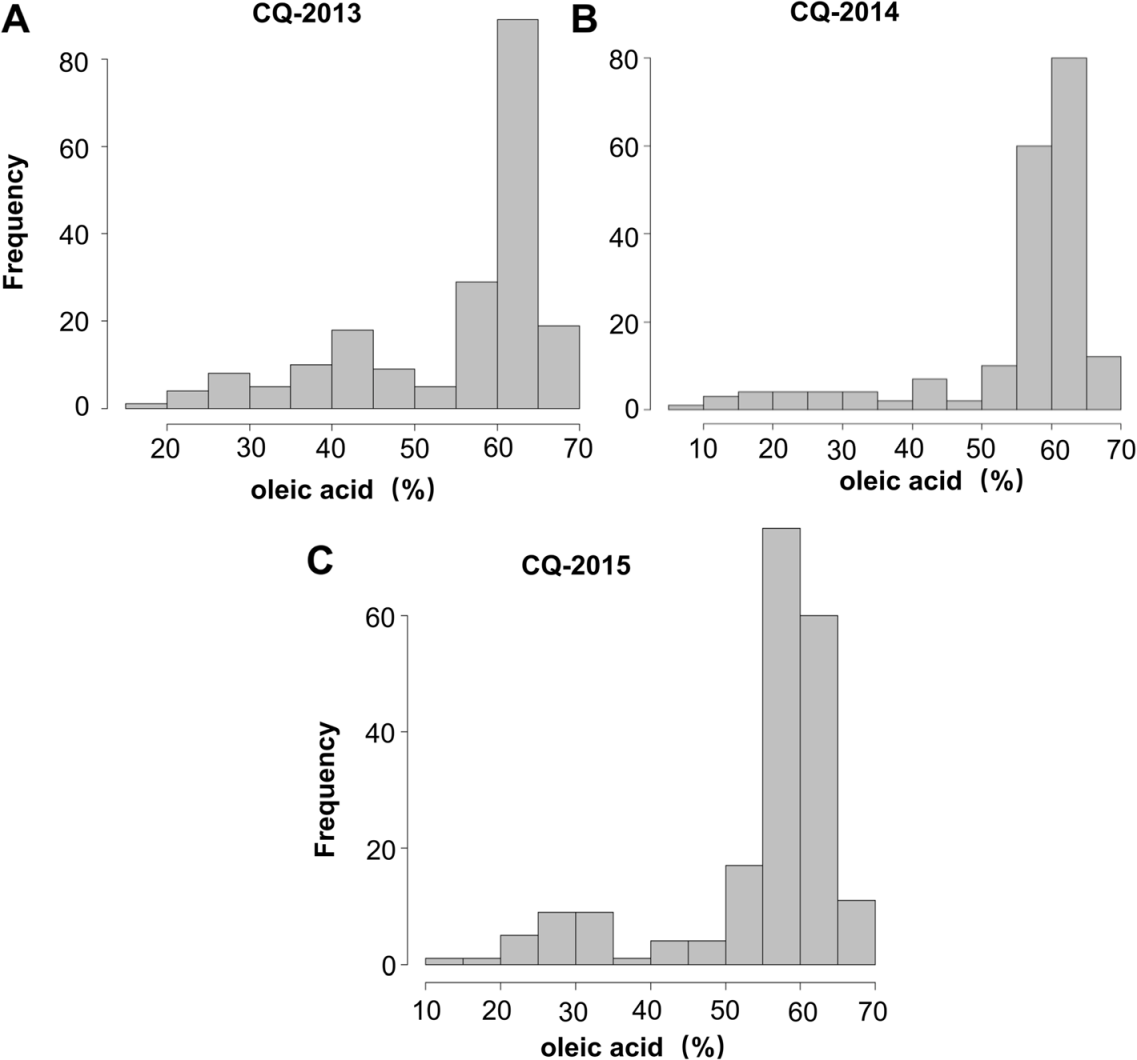
The frequency distribution of the oleic acid content in the three environments in 203 Chinese semi-winter rapeseed accessions. (**a**) 2013 in Chongqing (CQ-2013); (**b**) 2014 in Chongqing (CQ-2014); (**c**) 2015 in Chongqing (CQ-2015)

In this study, significant variation in the oleic acid content was observed across the diversity panel in three different years in Chongqing. The frequency distribution of the oleic acid content for the three different years is summarized in Fig. 1*.* In the three environments (years 2013, 2014 and 2015) in Chongqing, the oleic acid contents ranged from 19.83 to 67.83 (%), 6.49 to 67.20 (%) and 13.77 to 69.96 (%), with an average value (±SD) of 54.86 ± 12.1 (%), 55.53 ± 12.8 (%) and 54.74 ± 11.7 (%) and variable coefficients of 22.03, 22.99 and 21.32%, respectively *(*Table 1*)*. A high broad-sense heritability of H^*2*^ = 0.54 was calculated for the oleic acid content (Additional file 1: Table S1). The oleic acid content showed significant positive correlations across different environments (i.e. years) in Chongqing with a correlation coefficient of 0.42 to 0.72 (Table 1). This observation indicated that the oleic acid content exhibited relatively stable genetic variation in the diversity panel.

**Table 1:** Phenotypic characteristics of the oleic acid content in 203 Chinese semi-winter rapeseed accessions

Environment	Min (%)	Max (%)	Mean ± SD (%)	CV%	Environment	
					CQ-2014	CQ-2015
CQ-2013	19.83	67.83	54.86 ± 12.10	22.03	0.42***	0.55***
CQ-2014	6.49	67.20	55.53 ± 12.80	22.99		0.72***
CQ-2015	13.77	69.96	54.74 ± 11.70	21.32		

*CQ* Chongqing; *SD* standard deviation; *CV* coefficient of variation; ***p ≤ 0.001

### Oleic acid content GWAS

**Fig. 2:**
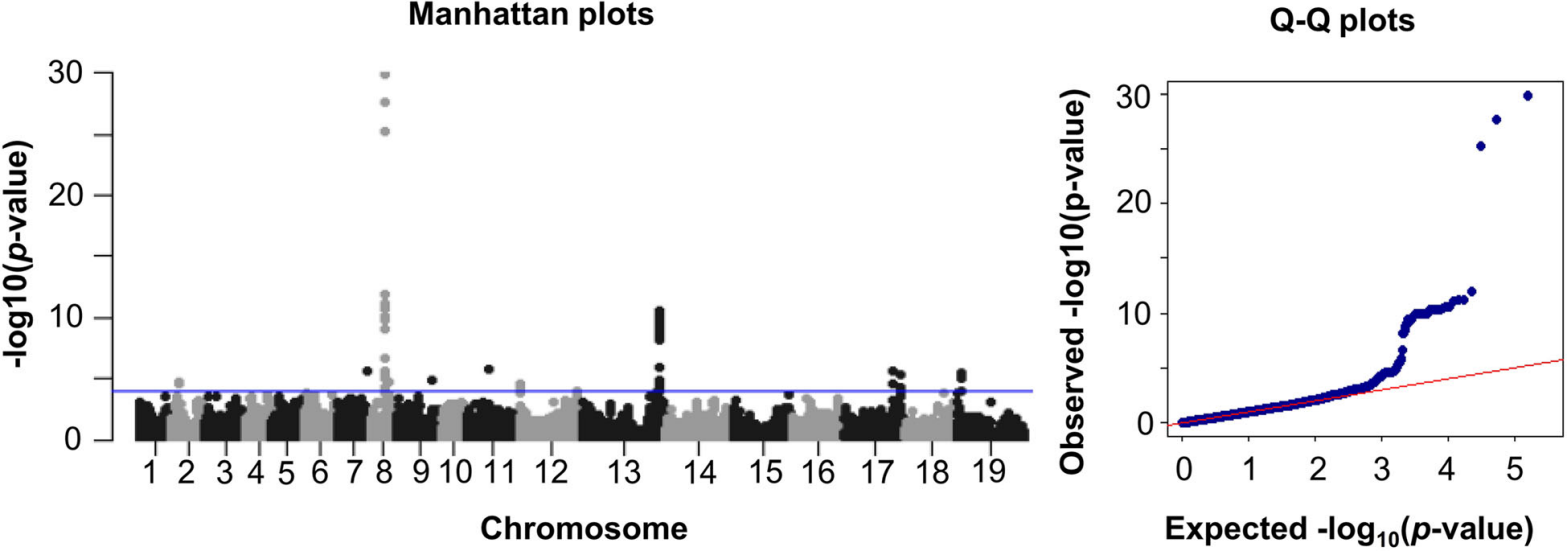
Manhattan and quantile-quantile (QQ) plots of MLM showing genome-wide marker-trait associations for oleic acid content in 203 Chinese semiwinter rapeseed accessions grown in three different environments. The –log_10_^(*p*)^ significance threshold of 4 is indicated with a horizontal blue line

**Fig. 3:**
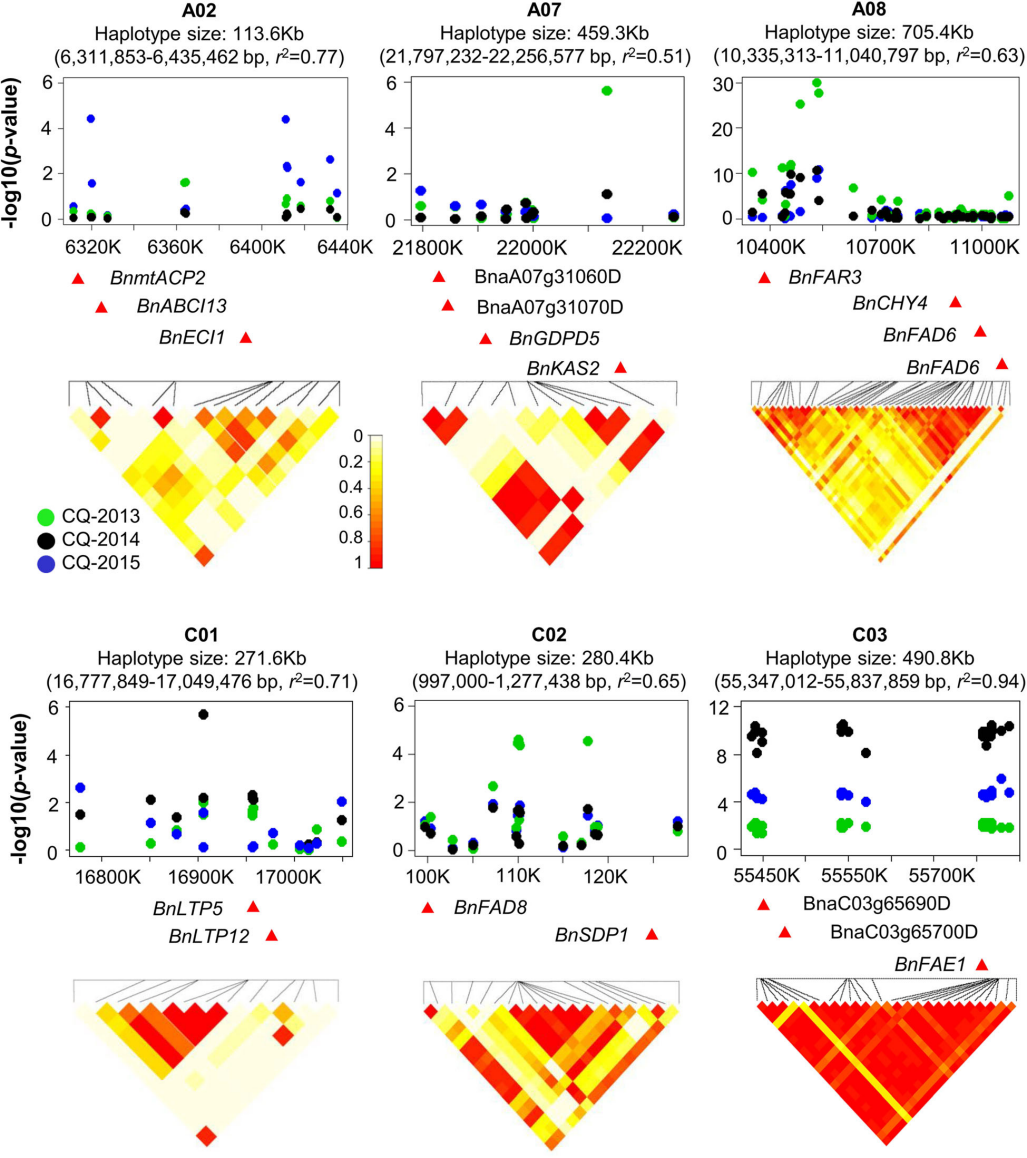
Six haplotype regions from chromosomes A07, A08, C01, C03, C07 and C09 carrying candidate genes that are significantly associated with the oleic acid content in Chinese semi-winter rapeseed accessions. The heatmap spans the SNP markers in LD with the most strongly associated SNPs

Manhattan plots and quantile–quantile plots describing significant SNP associations with the oleic acid content in the three different environments are shown in Fig. 2. A total of 51 SNPs distributed throughout the genome were detected in an association with oleic acid content using the significance threshold of –log_10_^(p)^ = 4. Candidate regions containing SNPs associated with the oleic acid content were investigated at a high resolution by assaying haplotype blocks (r^2^ > 0.50) in flanking chromosome segments. Six significantly associated haplotypes were detected on chromosomes A02, A07, A08, C01, C02 and C03 (Fig. 3)*.* Details of the SNPs and the candidate genes in the haplotype blocks with significant associations with oleic acid are shown in Additional file 2: Table S2.

### Multiple genes in close linkage with a positive effect on the accumulation of oleic acid content

**Fig. 4:**
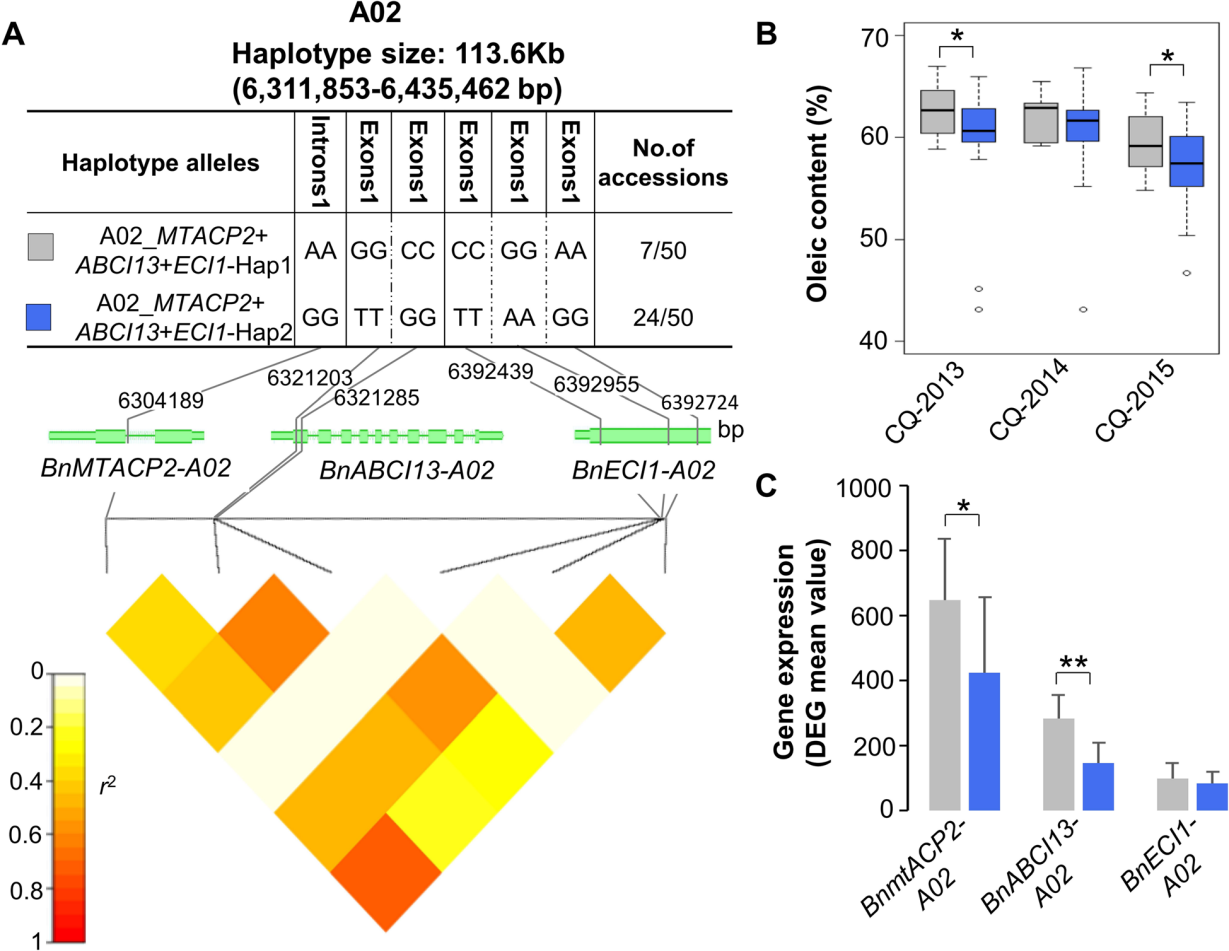
Detailed analysis of significant associations among the haplotype region (*6,311,853-6,435,462 bp, r*^*2*^ *= 0.77; A02_Hap*) in the A02 chromosome based on whole-genome sequencing of 50 Chinese semi-winter inbred lines. (**a**) High-density SNPs include six SNPs located in these three gene regions, including one in intron 1 of BnMTACP2-A02, two in exon 1 of BnABCI13-A02 and three in exon 1 of the BnECHIC-A02. (**b**) and (**c**) Two haplotype alleles with frequencies greater than 0.01 were identified in the haplotype region. The boxplots show that A02_ *BnMTACP2 + BnABCI13 + BnECI*_HAP1 has a higher oleic acid content and expression level than A02_ *BnMTACP2 + BnABCI13 + BnECI*_HAP2. *p ≤ 0.05, **p ≤ 0.01

On chromosome A02, we identified a haplotype block (6,311,853-6,435,462 bp; A02_Hap) significantly associated with oleic acid content, and containing three *B. napus orthologues of the Arabidopsis genes* MITOCHONDRIAL ACYL CARRIER PROTEIN 2 (BnMTACP2-A02; BnaA02g12050D), ATP-BINDING CASSETTE I13 (BnABCI13-A02; BnaA02g12080D) and DELTA (2)-ENOYL COA ISOMERASE 1 (BnECI1-A02; BnaA02g12140D) in this haplotype region (Fig. 3)*.* mtACP2 encodes a member of the mitochondrial acyl carrier protein (ACP) family that is involved in the fatty acid biosynthesis process, ABCI13 is an ATP-binding cassette (ABC) transporter that is involved in chloroplast lipid import, and ECI1 encodes a peroxisomal delta3, delta2-enoyl CoA isomerase that is involved in unsaturated fatty acid degradation (Additional file 2: Table S2). In addition, whole-genome resequencing of 50 accessions from the same diversity panel was used to further analyze the A02_Hap region. We found that six SNPs were located in these three gene regions, including one in intron 1 of BnMTACP2-A02, two in exon 1 of BnABCI13-A02 and three in exon 1 of the BnECHIC-A02 (Fig. 4a)*.* This result suggests that these three genes are in close linkage in the A02_Hap region. By comparing the oleic acid content and gene expression levels of the two haplotype alleles in the A02_Hap region, we found that A02_MTACP2+ ABCI13 + ECI1-HAP1 had a higher oleic acid content and that the BnABCI13-A02 and BnMTACP2-A02 genes also showed relatively higher expression levels than A02_MTACP2+ ABCI13 + ECI1-HAP2 (t-test and mean value; Fig. 4b and c*;* Additional file 3: Table S3).

*A* similar analysis conducted in the C02 chromosome region, haplotype block (997,000-1,277,438 bp; C02_Hap) was found to be significantly associated with oleic acid content (Fig*.* 3). This haplotype region contains three *B. napus orthologues* of the Arabidopsis genes FATTY ACID DESATURASE 8 (BnFAD8-C02; BnaC02g02240D) and SUGAR-DEPENDENT1 (BnSDP1-C02; BnaC02g02780D). FAD8 is involved in the fatty acid biosynthesis process, and SDP1 encodes a triacylglycerol lipase that is involved in the triglyceride metabolic process (Additional file 2: Table S2). In the C02_Hap region, combined whole-genome sequencing analysis of 50 accessions detected 9 SNPs located in these two gene regions, including six and three in the BnFAD8-C02 and BnSDP1-C02 regions, respectively *(*Additional file 6: Figure S1A*).* By comparing the oleic acid content and gene expression levels of the three haplotype alleles in the C02_Hap region, we found that C02_FAD8 + SDP1-Hap1 was associated with a higher oleic acid content than C02_FAD8 + SDP1-Hap2 and C02_FAD8 + SDP1-Hap3 and that BnFAD8-C02 and BnSDP1-C02 downregulated C02_FAD8 + SDP1-Hap1 expression in a manner that was related to higher oleic acid content accumulation (t-test and mean value; Additional file 6: Figure S1B and C*;* Additional file 3*:* Table S3)*.*

### Co-expression network of genes from the haplotype regions

To provide additional context for the proposed functions of BnMTACP2-A02, BnABCI13-A02 and BnECI1-A02 from the A02_Hap region and BnFAD8-C02 and BnSDP1-C02 from the C02_Hap region, we constructed a co-expression network for these five genes using gene expression data from siliques of Chinese semi-winter rapeseed accessions. The networks of the three and two genes from the A02_Hap and C02_Hap regions, respectively, were relatively independent *(*Additional file 8: Figure S3A). Then, we performed GO pathway analysis to uncover genes co-expressed with BnMTACP2-A02, BnABCI13-A02 and BnECI1-A02 in the A02_Hap region and BnFAD8-C02 and BnSDP1-C02 in the C02_Hap region. We found that these regions were significantly enriched in genes involved in lipid metabolic processes, fatty acid metabolic processes, acyl glycerol metabolic processes and so on (Additional file 7: Figure S2)*.*

**Fig. 5:**
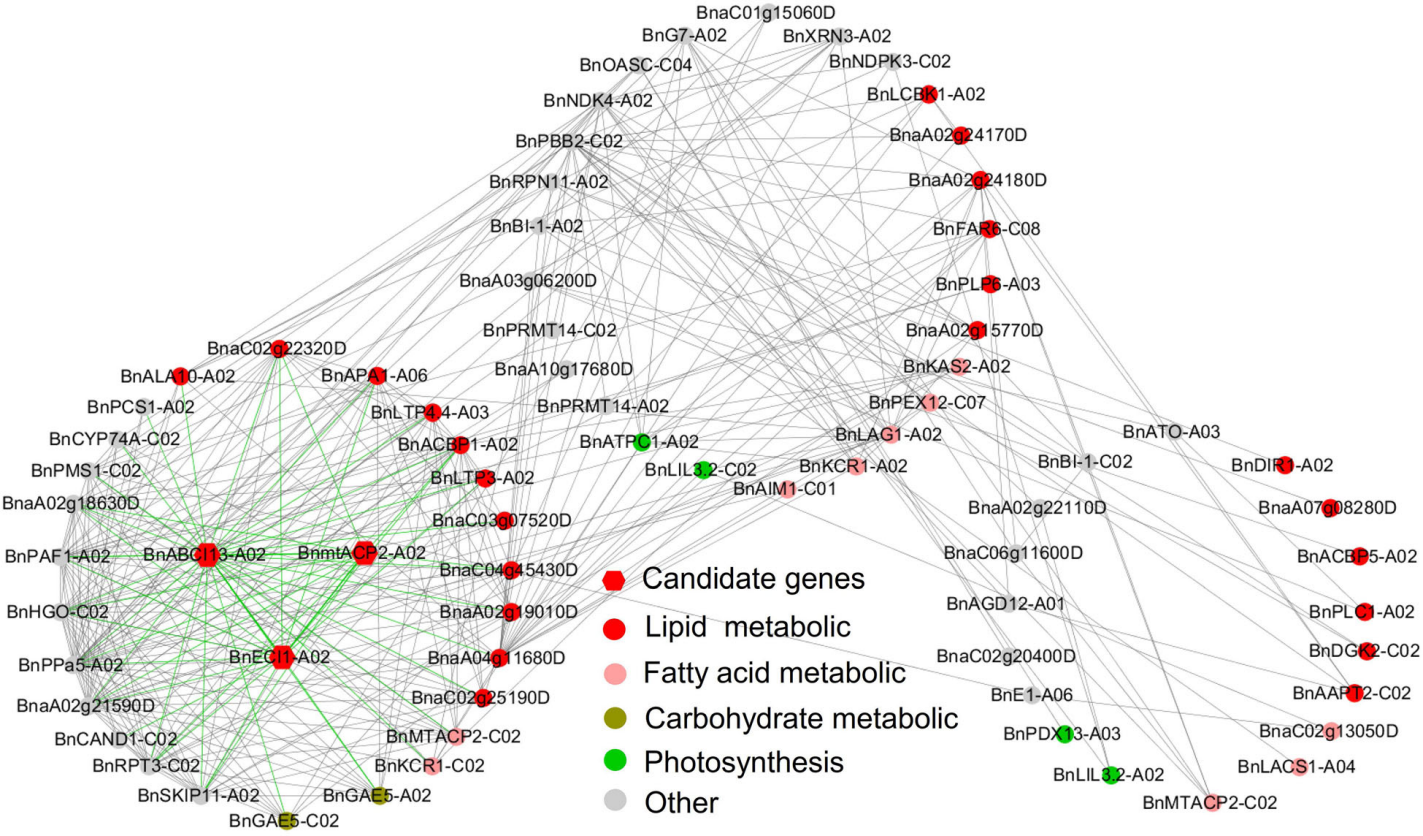
Co-expression network analysis of three candidate genes from the A02_Hap region. Red pentagon nodes represent the candidate genes *BnaMTACP2-A02, BnaABCI13-A02 and BnaECHIC-A02*. Based on the functional annotation, the co-expression network of these three candidate genes was classified into the following groups: lipids (red nodes), fatty acids (light pink nodes), glycerol (light goldenrod nodes), carbohydrates (yellow nodes), photosynthesis (green nodes) and others (grey nodes)

Based on the functional annotation, we further classified genes co-expressed with BnMTACP2-A02, BnABCI13-A02, and BnECI1-A02 from the A02_Hap region. These three candidate genes showed close correlations in the co-expression network (Fig. 5*;* Additional file 4: Table S4)*.* In these three candidate gene subnetworks, 7 (27%), 3 (12%), 5 (11%) and 2 (8%) genes were lipid-related, fatty acid-related, glycerol-related and carbohydrate-related, respectively. Another two clusters contained 12 (27%), 8 (18%) and 4 (9%) genes involved in lipid-related, fatty acid-related and photosynthesis processes, respectively *(*Fig. 5*;* Additional file 4: Table S4). These results showed that BnMTACP2-A02, BnABCI13-A02 and BnECI1-A02 from the A02_Hap region were interrelated with co-expression network genes that affected oleic acid content accumulation in rapeseed.

In the C02_Hap region, a similar gene co-expression network was constructed following this method. The candidate gene BnSDP1-C02 was indirectly related to the candidate gene BnFAD8-C02 in this network. In this co-expression network, a total of 14 genes were indirectly/directly associated with the candidate genes BnSDP1-C02 and BnFAD8-C02, including five and three genes involved in lipid and fatty acid metabolic processes, respectively (Additional file 8: Figure S3B*;* Additional file 4: Table S4)

## Discussion

Selection of high-quality rapeseed with increased oil content and improved edible oils with a modified fatty acid composition has always been an important breeding goal. Strong selection has caused the formation of highly conserved haplotypes that harbor agronomically major and minor genes in QTL regions [19, 20]. With the advent of high-throughput SNP genotyping, genome-wide panels of SNPs allow haplotype-based GWASs to explore several causal genes if they are in close linkage and are associated with the complex traits of interest. In this study, we performed a genome-wide analysis of haplotype blocks associated with the oleic acid content*.* We identified six haplotypes that were significantly associated with the oleic acid content, each of which contained at least two genes related to lipid transport, fatty acid metabolism or glycerol metabolism. Fatty acid compositions are typical quantitative traits that are controlled by polygenic inheritance. We performed a preliminary screen of at least 2000 genes related to the fatty acid/lipid metabolic processes in the whole genome by annotation analysis of the *B. napus Darmor-bzh* reference genome (data not shown). A recent study suggests that QTL regions containing many important genes involved in the pathway of fatty acid/lipid metabolism-related to seed fatty acid composition in *Brassica napus* [21]. Indeed, strong selection has created haplotype regions carrying fatty acid metabolism genes in major QTLs for the erucic acid content on chromosomes A08 and C03 [22]*.*

Generally, complex traits in crops show polygenic/multilocus quantitative inheritance. Selection causes multiple interacting genes/alleles to change in the same fitness direction, at a similar evolutionary rate, and across the same time scale, to achieve a common phenotypic outcome. The haplotype-based analyses can capture potential interactions between SNPs/genes at a locus that affect phenotypic variation [23, 24]. Qian et al. [25] suggested that the haplotype region carrying several causal genes related to photosynthesis, chlorophyll degradation, flavonoid and proline biosynthesis processes that forms a potential interaction adjust in rapeseed growth and adaptation. Our results suggested that strong selection has caused close linkages among MTACP2-A02, ABCI13-A02, and ECI1-A02 in the A02_Hap region, with positive effects on the accumulation of oleic acid content. MTACP2 encodes a member of the mitochondrial acyl carrier protein (ACP) family that is involved in the fatty acid biosynthesis process [26]. ABCI13 is an ATP-binding cassette (ABC) transporter that is involved in chloroplast lipid import [27]. In plants, de novo fatty acid synthesis occurs in the plastid stroma, where acyl chains grow attached to the acyl carrier protein (ACP) and become available for lipid assembly mainly in the form of C16:0-and C18:1-ACP [4, 28]*.* All acyl chains are made in plastids and assembled either in the plastids or the endoplasmic reticulum; thus, they require fatty acid/lipid transport [29]. ABC transporters direct participation in lipid or fatty acid/acyl-CoA transport. De Marcos Lousa et al. [30] suggested that functional and physical interactions between the ABC transporter and the peroxisomal chain acyl-CoA synthetases were adjusted during the fatty acid/lipid metabolic processes. ECI1 encodes a peroxisomal delta3, delta2-enoyl CoA isomerase that is involved in fatty acid beta-oxidation [31]*.* Fatty acid beta-oxidation is an important catabolic process that is required for generation of acetyl-CoA and entry into the citric acid cycle. In plants, this process occurs predominantly within the peroxisomes, and therefore fatty acyl-CoAs must be imported from the cytosol by ABC transporters. These results suggest that ABC transporters participate in the fatty acid synthesis/beta-oxidation process. Interestingly, we also detected a direct correlation among MTACP2-A02, ABCI13-A02, and ECI1-A02 in the co-expression network. These three genes also directly or indirectly correlated with fatty acid synthesis/beta-oxidation, lipid transfer, carbohydrate and photosynthesis genes in the network adjusted for the fatty acid metabolic process (Fig. 5).

A similar result was produced in the Hap_C02 region, which contained two genes (FAD8-C02 and SDP1-C02) with close linkage that affected phenotypic variation in the seed oleic acid content. FAD8 was located in the chloroplast that is involved in the unsaturated fatty acid biosynthetic process [32]*.* Unsaturated fatty acids in seed lipids are catalyzed by fatty acid desaturases (FADs), FAD6 converts oleic acid (C18:1) to linoleic acid (C18:2), and FAD7/FAD8 catalyzes the conversion of linoleic acid (C18:2) to linolenic acid (C18:3). Li et al. [33] suggested that a higher temperature caused a significant reduction in the FAD7/FAD8 expression level, which would increase oleic acid (C18:1) and linoleic acid (C18:2) accumulation and reduce the linolenic acid (C18:3) content. Drastic changes in temperature result in plant adaptation, with modified polyunsaturated fatty acid concentrations in their membranes and storage lipids. SDP1 limits triacylglycerol accumulation in vegetative tissues of Arabidopsis [34]. Fan et al. [35] suggested that SDP1 played key roles in diverting fatty acids from membrane lipid synthesis toward β-oxidation through a transient triacylglycerol pool. Kim et al. [36] *suggested that* SDP1 deficiency generated by RNAi technology produced a notable increase in seed oil accumulation. Our result showed that BnFAD8-C02 and BnSDP1-C02 expression was downregulated in accessions with higher oleic acid contents. Meanwhile, the co-expression network directs correlations of BnPLIP3-C08 with BnFAD8-C02, BnSDP1-C02, BnGED1-C02, BnMED15_1, BnLRS-C05, and BnPRMT4B-C05, which include five genes involved in fatty acid synthesis/beta-oxidation (Additional file 8: Figure S3)*.* These results suggest that strong selection has caused the genes of fatty acid synthesis/ beta-oxidation pathways being in a potential interaction and co-regulation for the accumulation of oleic acid in rapeseed.

Various arguments advocating haplotype-based analysis rather than single-marker analysis have been proposed. Haplotype analyses also provide important insights into the history of both natural and artificial selection (breeding) and can give valuable guidance to breeders seeking to diversify crop gene pools. Crop breeding programs aim to improve populations by increasing the frequency of favorable alleles of traits. The identification of shifts in allele frequencies within the genome can be important information for breeders, since it alerts them to monitor specific haplotype alleles and can be used to design appropriate breeding strategies [37]*.* Recently, some research has shown favorable haplotype alleles related to improved cold tolerance in rice [38]*,* short stature or long inflorescence branches in sorghum [39] and drought tolerance in maize [40]*.* We also reveal the beneficial haplotype alleles A02_MTACP2 + ABCI13 + ECI1-Hap1 and C02_FAD8 + SDP1-Hap1, which contribute to high oleic acid content. This finding will provide an opportunity to combine these beneficial haplotype alleles and further improve the oleic acid content in rapeseed.

## Conclusion

The high-throughput genome-wide single nucleotide polymorphic (SNP) markers have been successfully used in association mapping for the dissection of complex agronomic traits. Moreover, these markers allow whole-genome scans to identify haplotype regions that are significantly correlated with complex traits variation. In this study, GWAS in combination with haplotype analysis has identified six haplotype regions significantly associated with oleic acid (C18:1) that mapped to chromosomes A02, A07, A08, C01, C02, and C03. In addition, whole-genome sequencing of 50 rapeseed lines revealed *t*hree genes in the A02 chromosome haplotype region and two *genes in the* C02 chromosome haplotype region that were closely linked to the oleic acid content phenotypic variation. These results will help to develop functional haplotype markers for the improvement of the oleic acid content in rapeseed.

## Methods

### Plant material and phenotypic data

A diverse panel of 203 homozygous Chinese semi-winter inbred lines broadly encompassed the allelic variability in the Asian *B. napus* gene pool. The materials *(*Additional file 1: Table S1) were obtained as self-pollinated seeds from Southwest University, Chongqing, China, where they represent part of a breeding program spanning genetic diversity from the broader Asian gene pool. This population has been used previously in genomic diversity analysis [22] and GWAS of the chlorophyll content [22]. Field trials were conducted by applying an *unreplicated* complete randomized design in Chongqing in 2013, 2014 and 2015 *(*designated CQ-2013, CQ-2014, and CQ-2015, respectively*).* Each line was grown in a three-row plot with 10 homozygous inbred plants in each row. Self-pollinated seeds were collected from each line. About 5 g of dry mature seeds of each line (selected three strains, and each strain measured two times) were analyzed for oleic acid (C18:1) content by near-infrared spectroscopy (NIRS; Foss NIR Systems Inc., USA), each line selected three strains, and each strain measured two times.

The phenotypic analysis results, including the phenotypic distribution, mean value, standard deviation, correlation coefficient, and minimum and maximum values of oleic acid from the 203 accessions, were calculated and analyzed with the R packages Hmisc [41] and Psych [42]. Variance components were estimated via restricted maximum likelihood- using the statistical software package SPSS Statistics for Windows Version 22.0 (IBM Corp., Armonk, NY, USA), and broad-sense heritability (H^*2*^) for the oleic acid content was calculated, across the environments and in each environment, using the following equation [43]*:*

$$ {\mathrm{H}}^2=\frac{\upsigma_{\mathrm{g}}^2}{\upsigma_{\mathrm{g}}^2+{\upsigma}_{\mathrm{e}}^2/n} $$

*where the genotypic and residual variance components are represented by* ${\upsigma}_{\mathrm{g}}^2$ *and* ${\upsigma}_{\mathrm{e}}^2$*, respectively, and estimates of the residual variance were divided by the number (environments) n*.

### Genotypic data

The 203 accessions were genotyped using the Brassica 60 K Illumina® Infinium SNP array (IlluminaInc., San Diego, CA, USA) by TraitGenetics GmbH (Gatersleben, Germany). We applied a filtering for single-copy BLAST hits that were physically assignable to the *B. napus “Darmor-bzh”* reference genome assembly version 4.1 [44] *(*http://www.genoscope.cns.fr/brassicanapus/data/*).* Only markers that exhibited at least 95% sequence identity and no gaps in their 50-bp probe sequence were retained, resulting in 28,698 unique, locus-specific SNPs. In an additional preprocessing step, all markers with more than 10% missing values and a minor allele frequency below 5% were excluded. A total of 24,338 high-quality, single-locus, SNP markers with a minor allele frequency (MAF) ≥ 0.05 were used for the GWAS *(*Additional file 5: Table S5)*.*

### Genome-wide association analysis

A detailed description of the genetic composition and population structure of the diversity set was provided by Qian et al. [22]. Relative kinship analysis was performed using the TASSEL 5.0 software [45]*.* Marker–trait associations were examined by applying the TASSEL 5.0 software with the Q + K mixed linear model as proposed by Yu et al. [46]*.* The mixed linear model was as follows:

$$ \mathrm{y}=\upmu +\mathrm{S}\upalpha +\mathrm{Pv}+\mathrm{Zu}+\mathrm{e} $$

The model was used to test associations between the SNPs and phenotypes, where y is the vector of phenotypic observations, μ is the overall mean, α is a vector of the fixed allelic effects associated with the SNP under investigation, v is a vector of fixed population effects, u is a vector of random genetic background effects, and e is the vector of residuals. The matrix *P* contains information on population structure, using the first five principal components as fixed factors to adjust the model for population stratification, while S and Z are incidence matrices relating y to a and u, respectively. The variances of the random effects u and e are assumed to be normally distributed with u *~ N (0,* ${\mathrm{G}\upsigma}_{\mathrm{g}}^2$*) and e ~ N (0,* ${\mathrm{R}\upsigma}_{\mathrm{g}}^2$*), where G is a 203 ×* 203 genomic relationship matrix and R is a 203 × 203 matrix in which the off-diagonal elements are 0 and the diagonal elements are the reciprocal of the number of phenotypic observations per individual. The G matrix was computed according to the first method proposed by VanRaden et al. [47]*.* Since every genotype was tested only once per environment and GWAS were performed for all the environment–trait combinations separately, R corresponds to the identity matrix I.

The observed *P* values from the marker-trait associations were used to display Q–Q and Manhattan plots using the R package qqman [48]*.* The critical *P-value* for assessing the significance of SNP-trait associations was calculated separately for the oleic acid content based on the false discovery rate (FDR) [49]*. An FDR < 0.05 was used to identify significant associations with the oleic acid content at a cut-off value of -log*_*10*_^*(P)*^ *= 4.*

### 50 accession resequencing analysis

Genomic DNA was extracted from fresh leaves of each plant using the cetyltrimethyl ammonium bromide (CTAB) method and sequenced using the Illumina Hiseq™ 4000 (Illumina Co., Inc., San Diego, CA, USA) with a 5X sequencing depth and 125-bp paired-end sequencing length*.* Library preparation and sequencing were carried out at the Biomarker Technologies Corporation (Beijing, China*).* SNPs detected among the accessions that have described by Dong et al. [50]. SNPs with a missing call rate > 0.6 were excluded. The remaining SNPs with missing call rates below 0.6 were filled using the software “beagle” v4.1 [51] *(*https://faculty.washington.edu/browning/beagle/beagle.html). SNP loci with a heterozygous rate greater than 25% and a MAF less than 0.05 were removed. Ultimately, we obtained 532,005 high-quality SNPs that were used to analysis LD among candidate genes on significant association haplotype region.

### Phenotypic correlations with haplotype diversity groups

Significant haplotype blocks were identified using the R package LD heatmap [52]*,* with haplotypes defined across regions of homozygous markers with LD (r^2^) > 0.50 between the first and last markers in the block. Haplotype alleles with a frequency > 0.01 were used for the comparative phenotype analysis. A two-sample t-test (assuming unequal variance) was used to test for significant phenotypic differences in the oleic acid content among the haplotype alleles.

### Gene content in the haplotype block regions

Annotated genes within the oleic acid content-associated haplotype regions were extracted from the *B. napus* Darmor-bzh reference genome v. 4.1 [44]*.* To verify the most likely gene functions, we determined annotations of the closest orthologous *Arabidopsis thaliana* genes by BLAST matching against the Arabidopsis genome database (http://www.arabidopsis.org/).

### Transcriptome sequencing

Thirteen of 50 resequencing accessions were selected for the sampling of siliques for transcriptome analysis fifteen days after pollination. These 13 accessions were selected based on different oil contents (i.e. 4 accessions with lower oil content, 4 with medium oil content and 5 with higher oil content). The siliques were immediately frozen in liquid nitrogen and stored at *− 80 °C prior to RNA* extraction. At least 3 μg of total RNA was used for each accession to construct paired-end sequencing libraries according to the manufacturer’s instructions (Illumina Inc.). Paired-end reads (125 bp) were evaluated using the Illumina HiSeq 2500 platform. The raw reads were filtered to remove sequencing adapters and low-quality reads (> 10% unknown bases or > 50% of bases with a quality < 10) and generate “clean” reads that were subsequently aligned to the Darmor-bzh reference genome v.4.1 [44] *using HISAT 2 (*http://ccb.jhu.edu/software/hisat/index.shtml). The gene expression levels were calculated using HTSeq 0.6.1 [53] *(*https://pypi.python.org/pypi/HTSeq*).* To screen differentially expressed genes (DEGs), we used a q-value < 0.05 and an absolute value of log2 (fold change) normalized to > 1 based on the R package DEGSeq v1.22.0 [54]*.*

### Co-expression analysis

The co-expression edges were calculated, and a soft threshold value of 0.9 was chosen in the WGCNA R package [55]*.* Genes with Pearson Correlation Coefficients (PCCs) greater than or equal to 0.55 were used for co-expression network construction by CYTOSCAPE3.6 [56]*.*

## Supplementary information

**Additional file 1 Table S1.** Source, population structure, 50 resequencing accessions, 13 transcriptomes accessions, heritability and oleic acid phenotype information of 203 Chinese semi-winter rapeseed accessions.

**Additional file 2 Table S2.** SNPs and gene information in the significant association of haplotype region.

**Additional file 3 Table S3.** Detailed analysis of two haplotype regions from chromosomes A02 and C02, including SNPs, genes and haplotype alleles related to oleic acid content variation in 50 resequencing accessions.

**Additional file 4 Table S4.** Gene information of co-expression network in A02 and C02 chromosome haplotype region.

**Additional file 5 Table S5.** Flanking oligonucleotide sequences, chromosomal positions (best BLAST hit) and genotype calls for the 24,338unique polymorphic SNPs used for the chromosome structure and LD analyses in the diversity panel of 203 *B. napus* inbred lines.

**Additional file 6 Figure S1.** Detailed analysis of the significant associations of the haplotype region (997,000-1,277,438 bp; C02_Hap) in chromosome C02 by whole-genome sequencing of 50 Chinese semi-winter inbred lines. (A) A total of 21 SNPs were located in these three gene regions, including six and three in the *BnFAD8-C02* and *BnSDP1-C02* gene regions, respectively. (B) and (C) Three haplotype alleles with frequencies greater than 0.01 were identified in the haplotype region. The boxplots show that C02_*BnFAD8 + BnSDP1_*HAP1 has a higher oleic acid content and lower expression level than C02_*BnFAD8 + BnSDP1_*HAP3. *p ≤ 0.05, **p ≤ 0.01, ***p ≤ 0.01.

**Additional file 7 Figure S2.** GO pathway of three and two candidate genes from the A02_Hap and C02_Hap region co-expression networks, respectively.

**Additional file 8 Figure S3.** Co-expression networks of three and two candidate genes from the A02_Hap and C02_Hap regions, respectively. (A) Red pentagon nodes represent candidate genes in the A02-Hap and C02-Hap regions. Green and blue nodes represent genes that are directly and indirectly co-expressed with the candidate genes in these haplotype regions, respectively. (B) Co-expression network of two candidate genes from the C02_Hap region. Red pentagon nodes represent the candidate genes *BnFAD8-C02* and *BnSDP1-C02*. Based on the functional annotation, these two candidate gene expression network were classified into the following groups: lipid (red nodes), fatty acids (lightpink nodes) and others (grey nodes).

## Data Availability

All data generated and results analyzed during this study are included in this article and its supplementary information. Transcriptome data used for co-expression network and gene expression analysis in Additional file 4: Table S4. *24,338 high-quality SNP markers* for GWAS in Additional file 5: Table S5. Resequencing 50 accessions of Chinese semi-winter rapeseed from NCBI under BioProject accession PRJNA358784.

## Abbreviations

GWAS: Genome-wide association study
LD: Linkage disequilibrium
QTLs: Quantitative trait locus
SNP: Single nucleotide polymorphism
ACP: Acyl carrier protein
ABC: ATP-binding cassette
DEGs: Differentially expressed genes
PCCs: Pearson correlation coefficients
FDR: False discovery rate
MAF: Minor allele frequency
WGCNA: Weighted Gene Co-Expression Network Analysis
