# Supplementary figures and images for: GWAS and co-expression network combination uncovers multigenes with close linkage effects on the oleic acid content accumulation in Brassica napus
Source: BMC Genomics. 2020 Apr 23;21:320. doi: 10.1186/s12864-020-6711-0 (PMC7181522; doi:10.1186/s12864-020-6711-0)

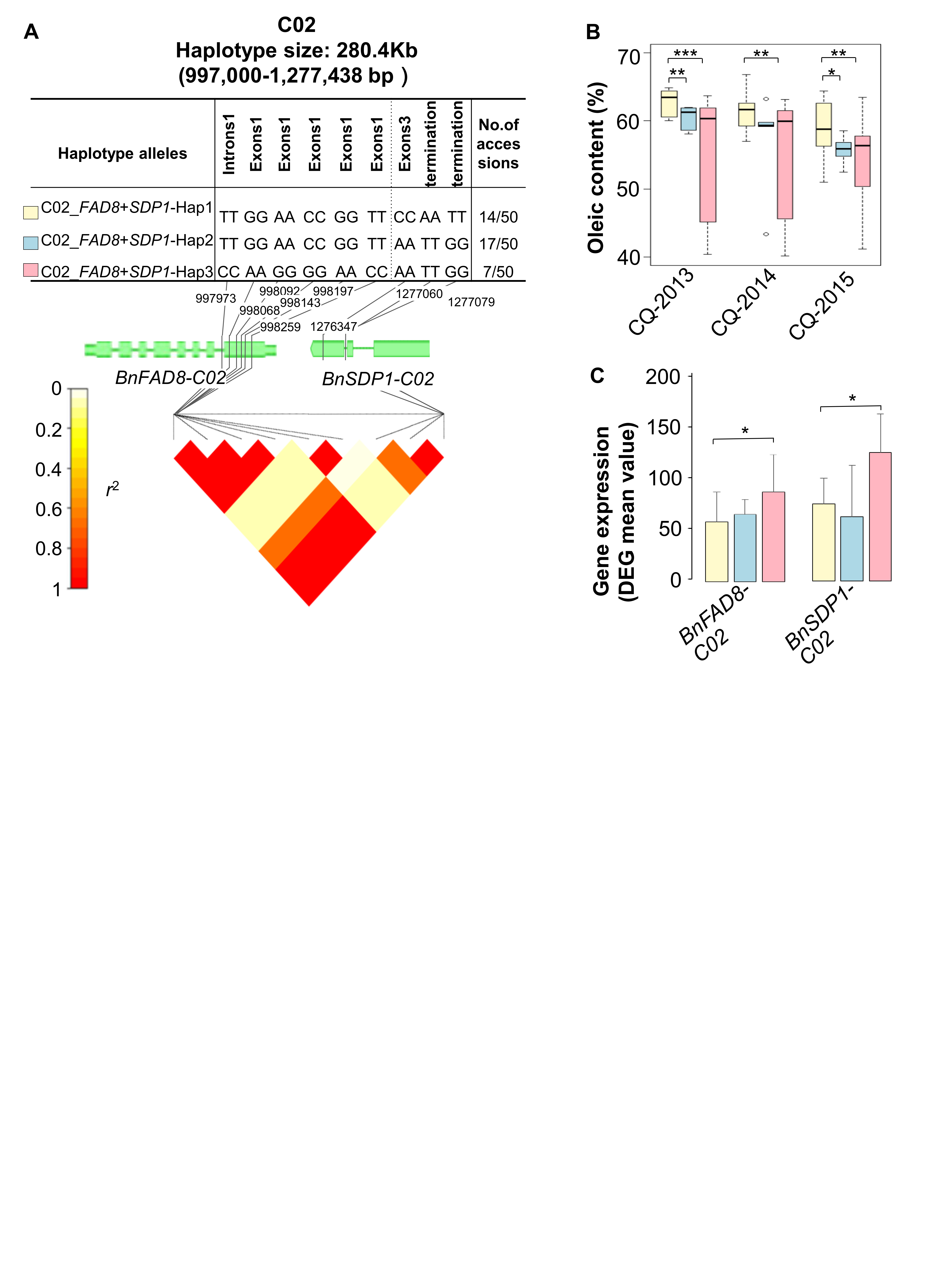

Supplement: Supplementary file 6 — Additional file 6 Figure S1. Detailed analysis of the significant associations of the haplotype region (997,000-1,277,438 bp; C02_Hap) in chromosome C02 by whole-genome sequencing of 50 Chinese semi-winter inbred lines. (A) A total of 21 SNPs were located in these three gene regions, including six and three in the BnFAD8-C02 and BnSDP1-C02 gene regions, respectively. (B) and (C) Three haplotype alleles with frequencies greater than 0.01 were identified in the haplotype region. The boxplots show that C02_BnFAD8 + BnSDP1_HAP1 has a higher oleic acid content and lower expression level than C02_BnFAD8 + BnSDP1_HAP3. *p ≤ 0.05, **p ≤ 0.01, ***p ≤ 0.01. [file 12864_2020_6711_MOESM6_ESM.tif]

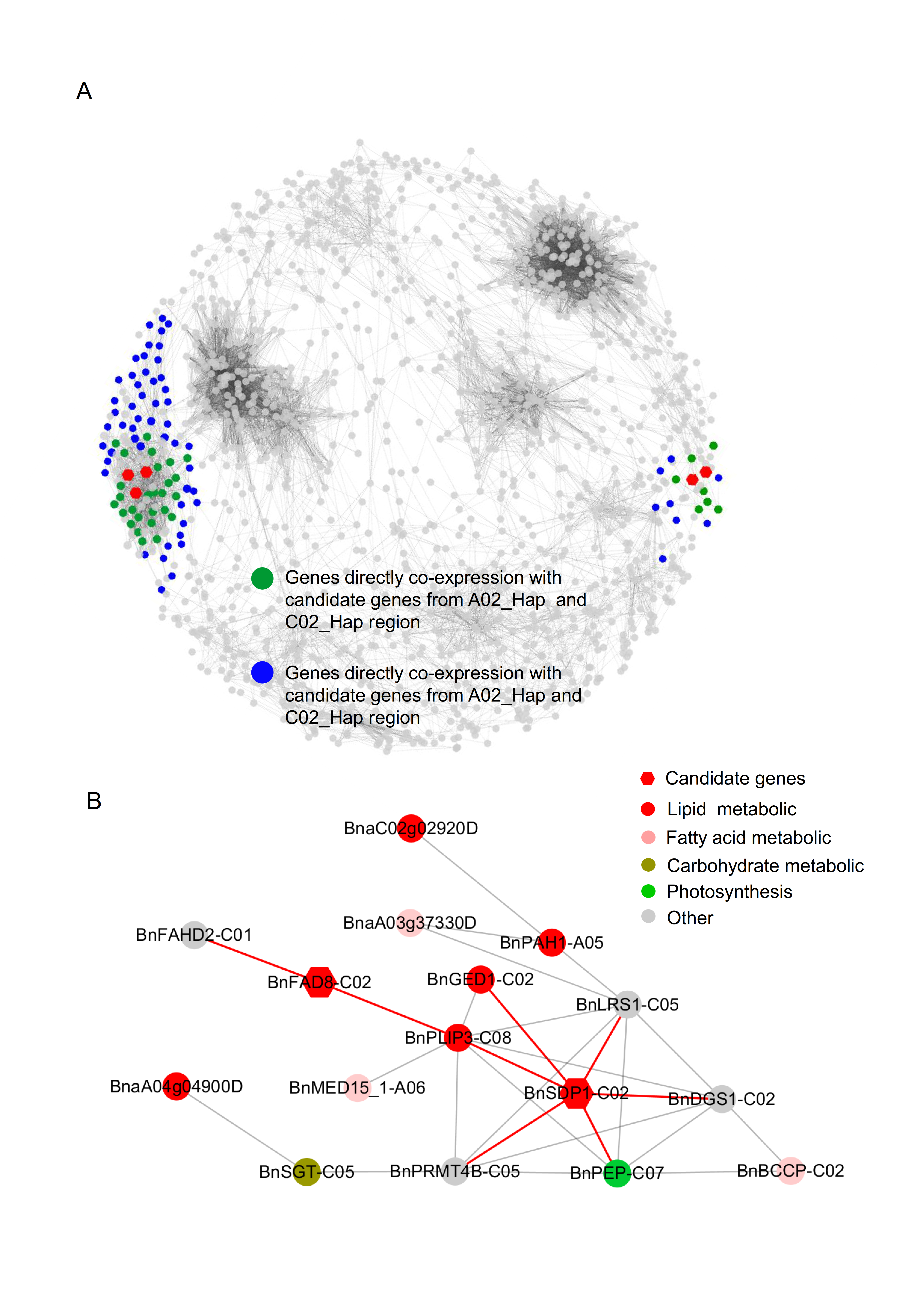

Supplement: Supplementary file 8 — Additional file 8 Figure S3. Co-expression networks of three and two candidate genes from the A02_Hap and C02_Hap regions, respectively. (A) Red pentagon nodes represent candidate genes in the A02-Hap and C02-Hap regions. Green and blue nodes represent genes that are directly and indirectly co-expressed with the candidate genes in these haplotype regions, respectively. (B) Co-expression network of two candidate genes from the C02_Hap region. Red pentagon nodes represent the candidate genes BnFAD8-C02 and BnSDP1-C02. Based on the functional annotation, these two candidate gene expression network were classified into the following groups: lipid (red nodes), fatty acids (lightpink nodes) and others (grey nodes). [file 12864_2020_6711_MOESM8_ESM.tif]
